# Autoencoder-based multimodal prediction of non-small cell lung cancer survival

**DOI:** 10.1038/s41598-023-42365-x

**Published:** 2023-09-22

**Authors:** Jacob G. Ellen, Etai Jacob, Nikos Nikolaou, Natasha Markuzon

**Affiliations:** 1 Institute of Health Informatics, University College London, London, UK; 2 AstraZeneca, Oncology Data Science, Waltham, MA USA

**Keywords:** Computational science, Non-small-cell lung cancer, Cancer models

## Abstract

The ability to accurately predict non-small cell lung cancer (NSCLC) patient survival is crucial for informing physician decision-making, and the increasing availability of multi-omics data offers the promise of enhancing prognosis predictions. We present a multimodal integration approach that leverages microRNA, mRNA, DNA methylation, long non-coding RNA (lncRNA) and clinical data to predict NSCLC survival and identify patient subtypes, utilizing denoising autoencoders for data compression and integration. Survival performance for patients with lung adenocarcinoma (LUAD) and squamous cell carcinoma (LUSC) was compared across modality combinations and data integration methods. Using The Cancer Genome Atlas data, our results demonstrate that survival prediction models combining multiple modalities outperform single modality models. The highest performance was achieved with a combination of only two modalities, lncRNA and clinical, at concordance indices (C-indices) of 0.69 ± 0.03 for LUAD and 0.62 ± 0.03 for LUSC. Models utilizing all five modalities achieved mean C-indices of 0.67 ± 0.04 and 0.63 ± 0.02 for LUAD and LUSC, respectively, while the best individual modality performance reached C-indices of 0.64 ± 0.03 for LUAD and 0.59 ± 0.03 for LUSC. Analysis of biological differences revealed two distinct survival subtypes with over 900 differentially expressed transcripts.

## Introduction

Lung cancer is the leading cause of cancer-related mortality worldwide, accounting for 18.4% of total cancer deaths in 2020^1^. Non-small cell lung cancer (NSCLC) makes up 85% of all cases, most of which fall into two major variations: lung adenocarcinoma (LUAD) and lung squamous cell carcinoma (LUSC), which comprise 40% and 30% of all lung cancers, respectively.

The ability to accurately project NSCLC prognosis is crucial to inform physician decision-making, but it remains a difficult prediction task. Traditional approaches tend to use unimodal data sources, most commonly clinical^2, 3^ or gene expression data^4, 5^, to predict survival. However, recent advances in high throughput “omics” technologies have led to publicly accessible descriptions of the genetic, epigenetic, and transcriptional profiles of cancer cells. Due to the high heterogeneity of lung cancer cells, the integration of multiple data types can offer improved survival analysis over unimodal approaches by providing more context for predictions. Although these multimodal datasets provide the potential to build more precise models of lung cancer progression, multimodal data integration techniques are still in their infancy^6^.

Many current multimodal approaches to predicting cancer progression are geared toward “pan-cancer” prediction with the use of deep learning in processing the data^7, 8^. Multimodal approaches for NSCLC prediction specifically tend to focus on either identifying clinically meaningful subtypes of patients, predicting patient survival^9^, or both^10^. For example, Lai et al.^9^ implemented a neural network that combined gene expression and clinical data to predict 5-year NSCLC survival. Another approach combined messenger RNA (mRNA), microRNA (miRNA), and clinical data to subtype LUAD patients before performing survival analysis and identifying survival-associated biological pathways^11^.

While emerging multimodal approaches are promising, they encounter a few common issues, including how to most effectively integrate heterogenous data modalities. Early integration, defined as omics concatenation prior to feature extraction, allows for the modeling of biological interactions between different omics types as the modalities are processed together. Conversely, late integration provides more precise representations of individual modalities, as each modality is extracted separately^12, 13^. The relative effectiveness of each approach depends heavily on the prediction task and the amount of data available^14^. However, early integration is more commonly used due to its potential to identify additional survival-related markers by combining multiple omics types^13^.

The most significant impediment to multimodal approaches remains the high dimensionality of sequencing data combined with the relatively small number of patients, causing models to overfit^6^. Solutions include reducing the dimensionality of the feature space by using supervised and unsupervised methods. Previously used feature extraction algorithms include principal component analysis (PCA)^15^, deep highway networks^7^, and autoencoders^16^. In particular, autoencoders, which are fully connected neural networks with “encoding” and “decoding” network paths^17^, have shown promise as a dimensionality reduction technique, including in application to cancer datasets^18^. They do so by reducing data dimensionality to a bottleneck in a hidden layer, while the decoding path uses the bottleneck representation to recreate the original input by minimizing a chosen loss function. Autoencoders have been found to improve accuracy in predicting kidney cancer survival compared to PCA by creating nonlinear feature combinations^16^.

Despite their demonstrated effective performance, autoencoders are highly prone to overfitting^17^. Recent survival prediction studies have experimented with denoising autoencoders^19^, which add excess noise to the input data to improve generalization to new data^20^. As an unsupervised method, autoencoders are not sensitive to features most relevant to survival prediction, which is problematic given that only a relatively small subset of genomic features contributes to survival prediction^5, 21^. One solution, which we implement here, is to use feature selection before denoising autoencoder compression to filter for the most relevant features.

In this study, we explored the benefits of multimodal data integration as applied to NSCLC survival analysis. This included the development of robust early-integration patient survival models that used a combination of feature selection and denoising autoencoders for dimensionality reduction. Using mRNA, miRNA, DNA methylation, long non-coding RNA (lncRNA), and clinical data, we aimed to demonstrate the advantage of combining multiple modalities of data in predicting NSCLC patient survival. To the best of our knowledge, this combination of modalities, and in particular the lncRNA modality in a multimodal context, have not been investigated for NSCLC survival prediction. We evaluated multiple data integration and parameter estimation methods, as well as different autoencoder variations to improve NSCLC survival prediction and to identify predictive biomarkers associated with patient survival.

## Results

### Performance of survival prediction in a multimodal setting

**Figure 1:**
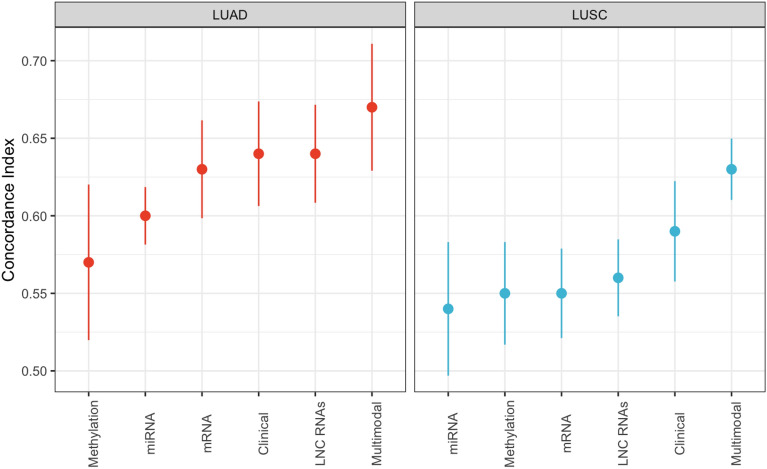
Predictive performance of survival models based on different modalities, including a multimodal approach, using independent test data. Both LUAD and LUSC models using multimodal data integration demonstrate improved performance as compared to models using individual modalities.

The predictive survival performance for models using each individual modality and their combination is shown in Fig. 1. Results were averaged across 5 independent test set evaluations (see ‘Training and evaluation approach’ section in Methods**)**. For multimodal models, results are reported using a single denoising zeros autoencoder (early integration), and an elastic net model trained on both LUAD and LUSC data. Results of variations of the pipeline discussed in the Methods are presented as Supplementary tables and figures.

The performance of the models predicting LUAD patients’ survival was superior to that of LUSC patients across individual modalities and in the multimodal setting, which agrees with previous findings^8^. As expected, the clinical data for both LUAD and LUSC were strongly prognostic. Yet, models based on multimodal data outperformed models based on clinical data alone and any other individual omics modality. Specifically, models utilizing early integrated multimodal data achieved mean C-indices of 0.67 ± 0.04 and 0.63 ± 0.02 for LUAD and LUSC data, respectively, compared to C-indices of 0.64 ± 0.03 and 0.59 ± 0.03 for clinical data alone. However, this difference was not statistically significant.

The biological data seemed particularly informative for LUAD, as all modalities except methylation achieved a C-index of at least 0.60. This is in contrast to the LUSC data, where all biological modalities except lncRNA scored at or below an average 0.55 C-index. Across both NSCLC subtypes, methylation, and miRNA showed the lowest average performance and appeared to be the least predictive modalities. Gene expression data were moderately predictive and performed best on LUAD data, scoring a mean C-index of 0.63 ± 0.03. Notably, lncRNA narrowly scored the highest of any individual biological modality for both LUAD and LUSC data, with mean C-indices of 0.64 ± 0.03 and 0.56 ± 0.02, respectively. Ultimately, two-sample T-testing between the mean C-indices of the individual modalities compared to the multimodal modality (both LUAD and LUSC) revealed varied significance with p-values of 0.04, 0.05, 0.08, 0.15 and 0.55 for the comparison of mean C-index of DNA methylation, miRNA, mRNA, lncRNA and clinical, respectively, to multimodal.

**Figure 2:**
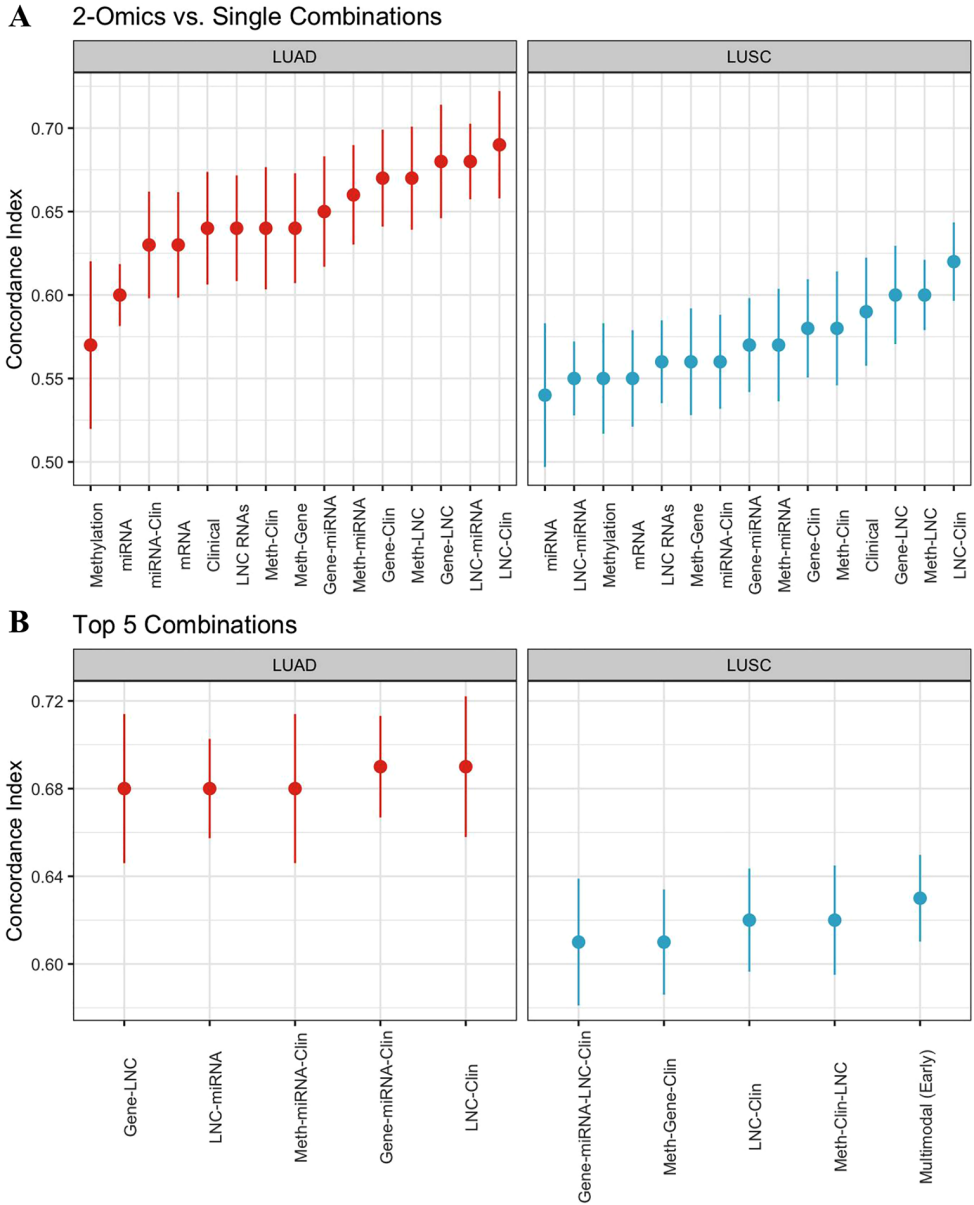
(**A**) Comparison of models’ performance using 2-omic combinations versus single modalities. (**B**) The top five highest-performing combinations of modalities. All models are evaluated on independent test data using mean C-index.

We evaluated combinations of modalities, as well as the relationships between modalities, to examine which among them were necessary for strong survival performance (Supplementary Table S1). Even combinations of only two modalities noticeably increased performance, as all 2-omics LUAD combinations (except miRNA and clinical data) outperformed every unimodal model (Fig. 2A). The LUSC results were similar except for clinical data, which was still one of the stronger predictors.

The top five combinations of modalities by mean C-index are shown in Fig. 2B. Notably, the combination of lncRNA and clinical data achieved the joint highest performance for LUAD, with a mean C-index of 0.69 ± 0.03, as compared with 0.67 ± 0.03 for all modalities. For LUSC, lncRNA and clinical data achieved a mean C-index of 0.62 ± 0.03, the second-highest result after early integration of all modalities. These relationships were not purely additive, as lncRNA showed only slightly higher performance than mRNA, but lncRNA and clinical data complemented each other well. Consistent with previous observations, all five highest-performing LUSC combinations contained clinical data. The combination of lncRNA and clinical data upon two-sample T-testing across both NSCLC types, achieved p-values of 0.03, 0.03 and 0.05 compared to the mean C-indices of DNA methylation, miRNA and mRNA data, respectively. However, a p-value of 0.11 was found in the comparison of the combination of clinical and lncRNA data to unimodal lncRNA and a p-value of 0.48 when comparing to the mean C-index of unimodal clinical data.

Although most high-performing modality combinations contained clinical data (Fig. 2B), the early integration of biological modalities as input improved predictive power for both LUSC and LUAD (Supplementary Table S1) over models using corresponding individual modalities. In fact, for LUAD, every early integration combination of biological modalities achieved equal or better performance compared to late integration. Strikingly, the early integration of LUAD methylation and miRNA data resulted in a 0.09 increase in mean C-index (from 0.57 ± 0.02 to 0.66 ± 0.03) compared with late integration. Average performance was also slightly higher for LUSC biological combinations when using early integration (0.013 mean difference). However, mean performance on data combinations that included clinical data was similar for LUAD and LUSC regardless of the time of integration (Supplementary Table S1).

### Combination of modalities for biomarker identification

#### Clustering analysis

**Figure 3:**
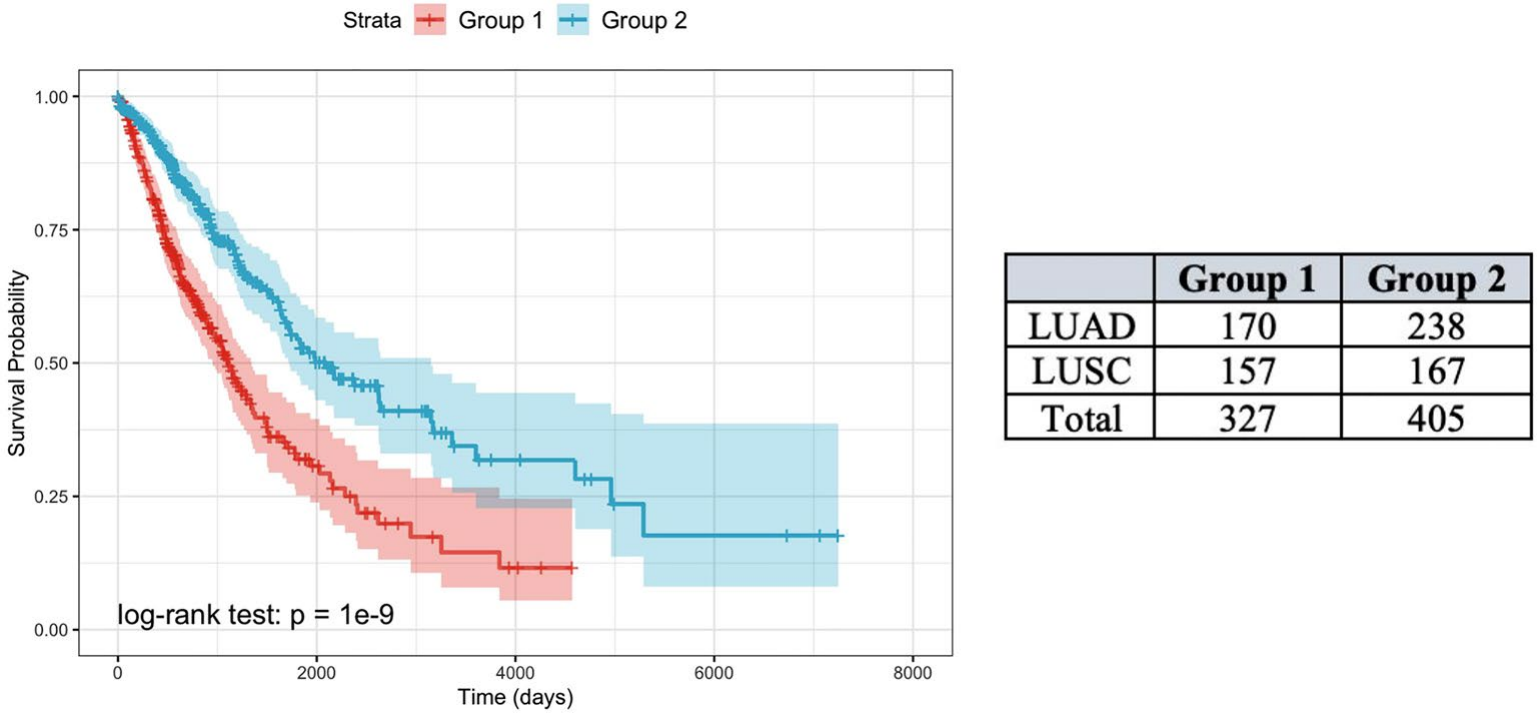
Survival differences between two groups of patients identified by K-means clustering based on multimodal feature space analysis.

K-means clustering was performed on the multimodal feature space using the multimodal non-zero features from the elastic net. The Silhouette method^22^ was used to identify the optimal cluster number, and the largest average silhouette width appeared at K = 2 clusters. A Kaplan–Meier curve showed a significant difference in survival between the two groups (Fig. 3; log-rank *P* = 1e-9). Clusters were better distinguished by survival outcome than by NSCLC type (both not participating in the clustering process), as there was a relatively even split of LUAD and LUSC cases in each cluster (Cluster 1, 52% LUAD cases; Cluster 2, 59% LUAD cases). Patients from Cluster 1 exhibited consistently lower survival times, which explains its slightly larger proportion of LUSC cases. Genes commonly linked to NSCLC survival, including *EGFR* (*P* = 0.002; log2 fold change [log2FC] = 0.42), *MET* (*P* = 0.04; log2FC = 0.29), and *ERBB2* (*P* = 0.02; log2FC = –0.23), were differentially expressed in Cluster 1, but the *KRAS* gene was not (*P* = 0.17; log2FC = 0.11).

#### Differential expression analysis

We used these two groups to identify differentially expressed transcripts among all four biological modalities. In Cluster 1, which was associated with poorer survival, we identified 507 differentially expressed genes (114 upregulated, 393 downregulated), 110 differentially expressed lncRNAs (57 upregulated; 53 downregulated), and 7 differentially expressed miRNAs (3 upregulated, 4 downregulated), (Supplementary Fig. S1). A total of 367 genes were differentially methylated in Cluster 1 compared to Cluster 2; of these, 224 genes displayed significantly higher mean methylation and 143 showed decreased methylation.

The top five differentially expressed mRNAs, miRNAs, and lncRNAs by log2FC and the top five differentially methylated genes by mean M-value difference across survival subtypes are shown in Table 1. Of the most downregulated genes in Cluster 1, low albumin expression is known as a poor survival indicator in NSCLC patients^23^. Less is known about solute carrier gene *SLC13A2*, but it has been implicated in cellular survival during lung tumorigenesis^24^. Additionally, *SCGB1A1* is a determinant of the success of NSCLC radiation therapy and immune checkpoint inhibitor combination therapy^25^.

**Table 1:** Top five differentially expressed transcripts between survival subtypes sorted by largest absolute log2FC values or absolute mean M-value difference for methylation.

Omics type	Gene	Log2FC/mean difference	*P*	Reference
mRNA	*ALB*	– 2.39	1.11e-24	Stares et al.^23^
	*SLC13A2*	– 1.80	7.41e-13	Bauer et al.^24^
	*NFE4*	1.75	2.61e-13	Pan et al.^46^
	*SCGB1A1*	– 1.63	3.28e-11	Ban et al.^25^
	*MTND1P23*	– 1.60	2.70e-14	Zhou et al.^47^
lncRNA	*ERVH48-1*	2.06	1.21e-25	Qi et al.^26^
	*LINC01287*	1.69	3.74e-10	Zhang et al.^27^
	*AC089983.1*	1.25	8.60e-10	Li et al.^48^
	*FLJ22447*	1.09	1.63e-14	Ding et al.^28^
	*RP11-356K23.1*	– 1.06	2.12e-08	Lou et al.^49^
miRNA	*hsa-mir-4449*	0.76	1.96e-05	Yan et al.^50^
	*hsa-mir-184*	– 0.67	1.01e-03	Lin et al.^51^
	*hsa-mir-34c*	– 0.67	1.34e-04	Kim et al.^29^
	*hsa-mir-891a*	0.64	3.90e-03	Wan & Zheng^30^
	*hsa-mir-34b*	– 0.62	8.4e-04	Kim et al.^29^
DNA methylation	*CAPS*	0.65	4.85e-05	Pastor et al.^52^
	*SEPT9*	0.44	1.28e-04	Powrózek et al.^31^
	*PTPRF*	0.39	1.60e-04	Soulières et al.^53^
	*FAM125B*	– 0.35	7.76e-05	Wang et al.^33^
	*PIK3R2*	0.32	1.37e-04	Vallejo-Díaz et al.^54^

The lncRNA with the greatest log2FC between the two groups, *ERVH48-1*, has been used to construct a LUSC prognostic signature of seven lncRNAs^26^, whereas *LINC01287* promotes proliferation and prevents apoptosis of LUAD cells^27^. In previous research, *FLJ22447* was found to reprogram fibroblasts to promote the growth of oral squamous-cell carcinoma^28^.

Both *hsa-mir-34b* and *hsa-mir-34c* were found to be significantly downregulated in group 1, in agreement with previous findings showing that these miRNAs suppress LUAD tumor growth and that decreased expression of both conferred poorer survival^29^. Upregulated miRNA *hsa-mir-891a* was recently identified as a novel biomarker for NSCLC in a study showing that increased expression was positively associated with NSCLC metastasis^30^.

For differentially methylated genes, *SEPT9* promoter hypermethylation has been evaluated as a marker for early diagnosis of lung cancer^31^, and its hypermethylation has been associated with poor prognosis in multiple cancer types^32^. Furthermore, in one study in which a methylation nomogram containing 11 probes was created for LUAD prognosis prediction, two of these probes were located in the body of the *FAM125B* gene (cg12133048, cg13600632)^33^.

### Exploratory analysis to improve prediction of patient survival

#### Comparison of denoising autoencoder performance

The denoising zeros autoencoder recorded the highest mean survival performance with the sigmoid activation function and with the addition of zeros to 30% of the matrix (Supplementary Table S2). These parameters achieved an average C-index of 0.592 across all modalities, which was greater than the next-highest value of 0.590. The latter was achieved by the denoising Gaussian autoencoder with a ReLU activation function and a 0.1 SD. Overall, the denoising zeros autoencoder achieved slightly higher average survival performance, achieving a mean C-index of 0.585 ± 0.005 as compared with 0.582 ± 0.005 for the denoising Gaussian autoencoder and 0.582 ± 0.003 for the basic autoencoder.

#### Varying training data

We examined whether training on only one NSCLC data type improved survival performance by increasing the specificity of predictions. LUSC performance improved after training on both cancer types, with an increase in mean C-index of 0.02 (0.54 ± 0.01 to 0.56 ± 0.01) across all modalities (Supplementary Table S3). Clinical and methylation modalities recorded mean C-index increases of 0.05, whereas multimodal and miRNA data both recorded a mean C-index increase of 0.04. The improved performance of LUSC when training was conducted on both NSCLC subtypes is probably related to the noisy biological signals that were present in the LUSC dataset, as well as to the larger patient sample size.

After training on both data types, the average C-index for LUAD data increased by only 0.01 (0.62 ± 0.02 to 0.63 ± 0.01) with 0.02 increases in the multimodal and methylation modalities. Otherwise, performance appeared similar other than a 0.02 decrease for the miRNA modality (Supplementary Table S3).

#### Early vs. late data integration

It is unclear whether early or late multimodal integration of genomic data leads to better task performance^12^. In this analysis, early and late integration performed similarly for the LUAD dataset (Supplementary Table S4) while LUSC showed a striking 0.04 increase in mean C-index (0.59 ± 0.03 to 0.63 ± 0.02) when early integration was used. In this case, nonlinear combinations of LUSC biological features created by the autoencoder were clearly more predictive than features from individual modalities.

#### Evaluation of alternative dimensionality reduction schemes

The combination of feature selection and denoising autoencoder narrowly outperformed three alternative pipelines for LUAD data for both early and late integration (Supplementary Fig. S2). For LUSC late integration, the combined approach was less predictive than either feature selection or denoising autoencoders individually. Yet early integration decisively favored our proposed approach (mean C-index = 0.63 ± 0.02), achieving the highest overall LUSC mean C-index. Although the differences were not large, the combined approach achieved the highest survival performance, with LFS representing the next-best methodology.

## Discussion

Our results show that modality integration improved prognosis prediction, as using multimodal data increased survival discrimination over that of any individual modality. Furthermore, 2-omics combinations outperformed almost all unimodal approaches for both NSCLC types. These findings occurred despite the increased feature space dimensionality of modality combinations demonstrating the value of the proposed data integration methodology. Our findings are consistent with those of a previous liver cancer multimodal approach^16^ and provide strong evidence for the potential of multimodal data integration to better describe the heterogeneity of NSCLC patients.

Models based on a combination of clinical and lncRNA data achieved high performance for both LUAD and LUSC (mean C-indices of 0.69 ± 0.03 and 0.62 ± 0.03, respectively). Notably, lncRNAs were included in three and four of the five top-performing modalities combinations for LUSC and LUAD. This could partially be explained by the low correlation between lncRNA and other modalities. Further, although the investigation of lncRNAs in cancer is relatively new, lncRNAs are commonly dysregulated in NSCLC^34^. However, with the exception of one study that used lncRNAs and clinical data to establish a NSCLC risk score for binary survival^35^, to our knowledge no other multimodal studies have examined the predictive power of the lncRNA modality for both LUAD and LUSC together. Our study suggests the predictive value of this modality, with significant overlap in identified profiles for LUAD and LUSC. Further research is warranted to validate this finding.

Overall, the highest survival performance was achieved with lncRNA and clinical data (or gene expression, miRNA, and clinical data) for LUAD (mean C-index = 0.69 ± 0.03) and using all modalities for LUSC (mean C-index = 0.63 ± 0.02). It is challenging to directly compare survival performance with other studies because of differences in performance evaluation methods and the dataset utilized. A notable TCGA LUSC study implemented a multimodal approach by using basic autoencoders and four biological data types to achieve a C-index of 0.597^19^. Another study combined histopathology whole-slide images (WSIs) with clinical data for a 0.62 C-index^36^. In an examination of previous TCGA LUAD dataset performance, one study used mRNA data to achieve a C-index of 0.656^37^, and a second integrated mRNA and clinical data for a C-index of 0.689^38^. Finally, a LUAD study used an autoencoder to combine mRNA, miRNA, methylation, and copy-number variation data to achieve a 0.65 C-index^39^. As compared to the findings above, our proposed methodology demonstrates comparable or stronger performance using similar data and provides biologically actionable results. A higher performance achieved by Cheerla & Gevaert^7^ can potentially be explained by an increase in the training data size when using pan-cancer TCGA data (20 different cancer types), which is in line with our finding of improved performance when using both LUAD and LUSC data for training.

In addition to improved predictive power, our approach has demonstrated that multimodal analysis provides a unique opportunity to build robust survival subgroups that take multiple aspects of a patient’s biology and clinical status into consideration. Our differential analysis based on those survival subgroups identified a combination of differentially expressed mRNAs, miRNAs, and lncRNAs, as well as differentially methylated genes associated with improved or shortened survival. Top genes in these categories have been previously linked to shown to be associated with NSCLC and/or cancer prognosis (see ‘Differential expression analysis’ section of Results).

Several limitations were placed on data selection in this work. These include the selection of patients with all the modalities present, which could have introduced a bias in the patient population. Along these same lines, this approach must be further validated on additional external datasets as more and more of these multimodal datasets become publicly available in the future. In addition, we did not explore other data modalities, such as proteomic, copy-number variation, or WSI data, that could allow for more effective survival predictions. The lack of interpretability of autoencoders is also a major limitation of this study, as it is difficult to determine what the extracted dimensions of the reduced feature space represent.

## Methods

### Overview

**Figure 4:**
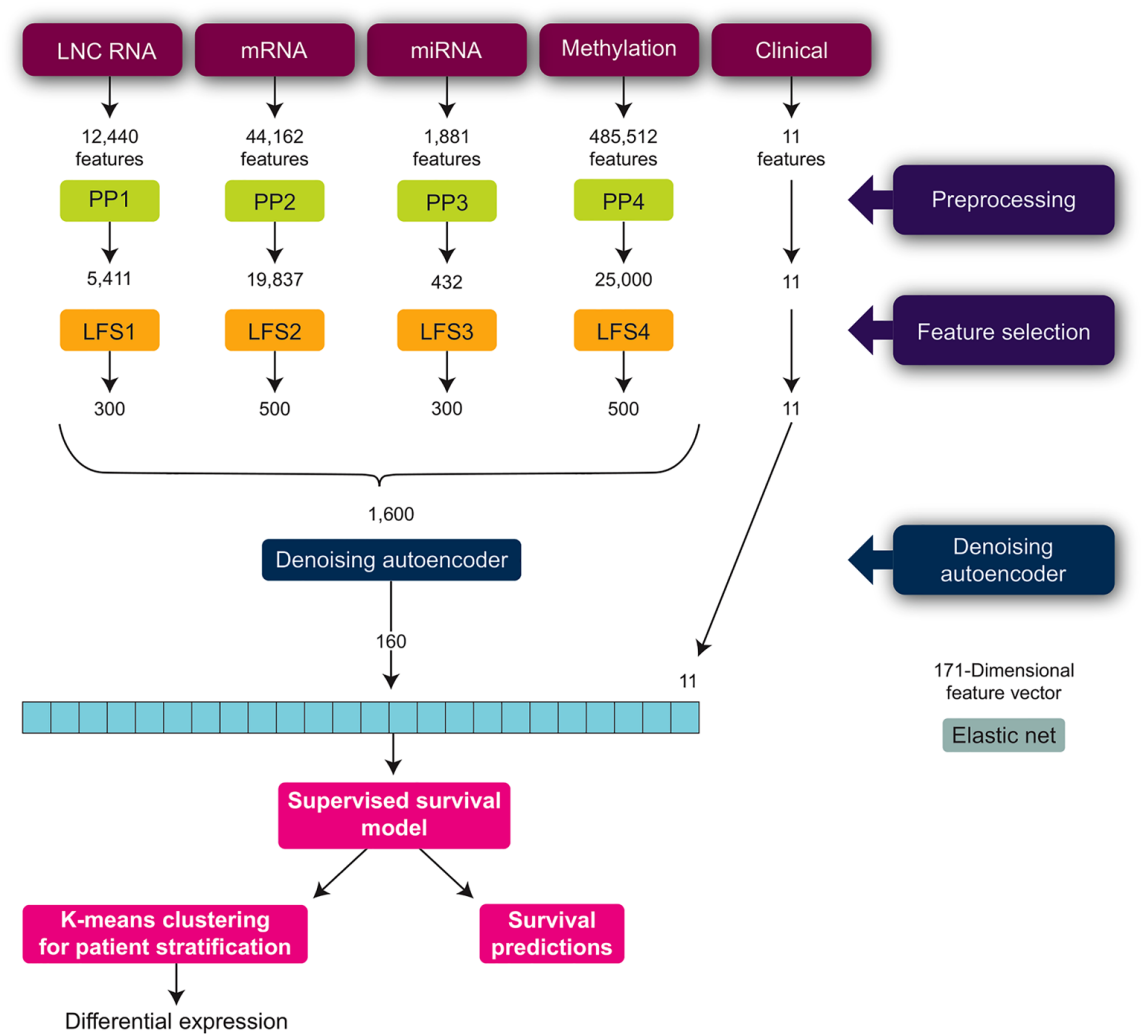
Proposed pipeline for NSCLC survival analysis.

Our proposed data analysis approach is summarized in Fig. 4 and includes:

- Preprocessing steps: Data cleaning and supervised linear feature selection were carried out to identify significant features and reduce the dimensionality of the input data. These steps were performed for each biological modality separately.
- Modality integration: A single autoencoder was used to integrate and compress biological data from all modalities together into a 160-dimensional feature space. Adding the clinical modality data brought the dimensionality of the input data to 171. Several types of autoencoders were evaluated for improved survival performance, with a demonstrated advantage of the denoising autoencoder.
- Survival analysis: We implemented the elastic net model, a regularized Cox proportional hazards (PH) model.
- Biomarker identification: K-means clustering was conducted on the survival-associated variables (as identified by the elastic net model) of the multimodal feature space to identify survival subtypes. Differential expression analysis was carried out between the survival subtypes to identify biological transcripts associated with survival outcomes.

### Data

Data for this study were obtained from The Cancer Genome Atlas (TCGA) dataset, which contains multiple modalities of lung cancer data, including 1881 miRNAs, mRNA expression data for 44,162 genes, DNA methylation data from 485,512 probes, and clinical data (Supplementary Table S5)^40^. In addition, 12,440 lncRNAs were extracted from the mRNA modality according to the lncRNA list published by Lin et al.^41^. Each patient has a time-to-death in days right-censored up to 11,000 days post-diagnosis and a binary survival status variable.

A total of 732 NSCLC patients had samples in all five modalities. We evaluated LUAD and LUSC subpopulations separately to better understand the differences and generalizable commonalities in subpopulations. Of these patients, 449 (61.3%) survived (or were censored) and 283 (38.7%) died during the study period.

After all data modalities were combined, there were 544,006 features in the initial feature space. For clinical data, we used 11 features provided by TCGA (Table 2). The “tumor volume” variable was calculated by computing the volume of the tumor using the three dimensions provided in the dataset.

**Table 2:** Clinical features included in TCGA dataset.

Clinical feature	Feature levels^a^	No. of missing samples
Age (years)	66.1 ± 9.5	14
Sex	Male	0
	Female	
Tumor volume (cm^3^)	0.5 ± 0.5	220
Primary diagnosis	Adenocarcinoma	0
	Squamous cell carcinoma	
Prior malignancy	Yes, no	0
Synchronous malignancy	Yes, no	54
Pathological stage	Stage1, Stage 1A, Stage 1B	8
	Stage 2, Stage 2A, Stage 2B	
	Stage 3, Stage 3A, Stage 3B	
	Stage 4	
Staging tumor	T1, T1a, T1b	3
	T2, T2a, T2b	
	T3	
	T4	
Staging lymph nodes	N0	12
	N1	
	N2	
Staging metastasis	M0	199
	M1, M1a, M1b	
No. of pack-years smoked^b^	46.2 ± 28.1	180

^a^Variable data are expressed as mean ± SD.

^b^A pack-year is defined as 20 cigarettes smoked every day for 1 year.

### Preprocessing

For the initial preprocessing steps, the LUAD and LUSC datasets were combined as has been reported previously^2, 10^. Individual mRNA, miRNA, and lncRNA features were removed if they had zero or missing values in more than 20% of patients in the dataset. Patients with follow-up times of 0 or 1 day were also removed. The features of each modality were Z-scored (i.e. each feature had zero mean and unit variance). Imputation was performed for missing values using the median. These computations were performed separately for training and testing data.

DNA methylation features in the TCGA dataset consist of beta values from 485,512 probes ranging from 0 to 1 based on methylation status. Probes with more than 20% missing values were excluded (remaining with 395,616 probes). In addition, only the 131,106 probes found in CpG islands within 1500 base pairs upstream of the transcriptional start site were used, a commonly used methylation processing approach^10, 39^. Finally, before LFS, the top 25,000 probes with the highest beta value variance were filtered to extract more informative features. This led to a reduction of lncRNA features from 12,440 to 5411, mRNA features from 44,162 to 19,837, miRNA features from 1881 to 432, and methylation features from 485,512 to 25,000 (Fig. 4).

### Linear feature selection (LFS)

Prior to autoencoder-based data integration and lower dimensionality projection, each feature in each modality was separately evaluated through an LFS process to ensure feature relevance to the prediction task. Specifically, Wald significance tests based on univariate Cox PH models were employed to measure the association of each feature with patient survival time. The top 500 features for mRNA and methylation and the top 300 features for lncRNA and miRNA with the lowest Wald *P* values were selected for each modality, proportional to the initial number of features in each modality. Feature selection was performed using training data only, with validation schema presented in the ‘Training and evaluation approach’ section below.

### Denoising autoencoder

The final NSCLC pipeline employs a single denoising “zeros” autoencoder, which adds noise to the input data by randomly replacing a percentage of the input matrix with zeros^42^. The denoising zeros autoencoder takes the concatenated biological data (1600 features) and reduces the data to a 160-dimensional vector (Supplementary Fig. S3). Before choosing our final autoencoder structure, we tested and compared the performance of this denoising zeros autoencoder to a denoising autoencoder that adds zero-centered Gaussian noise to the data with a chosen standard deviation (SD) of the distribution (denoising Gaussian autoencoder)^18^. We also compared performance to that of a basic autoencoder with no denoising function (no additional hyperparameters). Performance of these autoencoder models and hyperparameter combinations were assessed by averaging C-index across each individual modality as well as the multimodal modality (Supplementary Table S2).

All models employed an adaptive moment estimation optimizer, a mean squared error loss function, and a learning rate of 0.001 to balance accuracy and training time. To combat overfitting, all autoencoders were trained for 100 epochs with an early stopping mechanism that ended training if validation loss did not improve for 5 epochs. For each denoising autoencoder type, the rectified linear unit (ReLU), tanh, and sigmoid activation functions were compared. For the denoising zeros autoencoder, the proportion of matrix observations replaced with zeros was modulated (0.2, 0.3, 0.4) and the SD of the distribution of noise added (0.5, 1, 1.5) was adjusted for the denoising Gaussian model.

### Supervised survival modeling

For this study, we used an elastic net as the survival model. This model is a regularized form of the Cox PH model that deals effectively with high-dimensional data by preventing overfitting^43^. More specifically, elastic net uses both Lasso (L1) and Ridge (L2) regularization together to penalize complex Cox PH models and decrease coefficient values. The alpha value (between 0 and 1) determines how much weight is given to the L1 and L2 penalties, and an alpha value of 0.5 was chosen to give equal weight to each. Training data was used to find the optimal lambda value, a coefficient that determines the degree of coefficient shrinkage.

### Integration of different combinations of modalities

In addition to combining all the modalities in survival prediction models, we evaluated several two, three, and four modality combinations. Both early and late data integration were evaluated for each combination.

### Survival clustering stratification and differential expression analysis for biomarker identification

To gain additional insight into the features most affecting patient survival, we implemented K-means clustering using features significantly associated with survival by the elastic net model. By using one training run, features with coefficients driven to zero by the elastic net model were removed to keep only survival-associated features. These survival-related features were used in unsupervised K-means clustering to identify distinct survival subtypes. The optimal number of clusters was determined by silhouette index (2 to 10 clusters evaluated)^22^.

Using these identified survival subtypes, we conducted differential expression analysis to find differences in mRNA, miRNA, and lncRNA expression between groups. Differential expression was defined as a false-discovery rate adjusted *P* value of < 0.05 and absolute fold change of > 1.5.

For the methylation data, beta values were mapped to M-values through a logistic transformation^44^. Methylation probes were mapped to genes by averaging M-values across each gene. An analysis-of-variance test identified differentially methylated genes based on the mean M-value, as described previously (*P* < 0.05)^39^.

### Training and evaluation approach

We used a robust training/validation and independent test set schema for reporting the results. Training/validation data (80% of the data) were used for parameter estimation through all preprocessing and modeling steps. Results are reported on the independent test set (20% of the data). To further assess the stability of the results and to report confidence intervals, a bootstrapping schema was introduced. The results are reported across these 5 independent runs.

### Performance evaluation

To estimate prediction error, we used the Harrell concordance index (C-index), the most commonly used metric for survival analysis^45^. The C-index is used to evaluate the probability that a model will correctly predict which of two randomly selected patients will die first. In other words, it measures the ability to correctly order patients in terms of survival time, ranging from 0.5 (random chance) to 1 (perfect prediction).

### Exploratory analysis to improve patient survival prediction

#### Varying training data

Earlier studies showed improved performance of survival models when training using pan-cancer data^7^. However, due to the prevalence of LUAD over LUSC cases in TCGA, the survival model may prioritize features that are geared more toward LUAD, compromising LUSC performance if training is done on both LUAD and LUSC. We therefore compared models’ performance trained using LUAD or LUSC data only versus their combination.

#### Early vs. late data integration

The effect of reducing the data dimensionality of each data type separately (late integration) was compared with that of using one denoising autoencoder to compress all of the biological data into a 160-dimensional vector together (early integration). For late integration, each autoencoder was structured with layers of size 500, 50, and 500 for mRNA and methylation data or 300, 30, and 300 for miRNA and lncRNA data. Survival performance was compared by using the C-index of each integration scheme on multimodal data and all possible combinations of omics data.

#### Comparison with other dimensionality reduction approaches

The performance of this combined feature selection and autoencoder feature reduction approach was compared with three alternative data dimensionality reduction methods. These alternatives were a denoising autoencoder with no feature selection, PCA, and LFS with no autoencoder. We compared the survival performance of all four techniques on multimodal data across both NSCLC type and early and late modality integration.

### Implementation

All data analyses for this study were completed with R, version 4.2.0. The Keras package in R with a Tensorflow back end was employed to implement the denoising autoencoders. Differential expression analysis was implemented with the DESeq2 R package, and the lumi R package was used to map beta values to M-values for differential methylation analysis.

### Supplementary Information

Supplementary Information.

## Data Availability

The code used to generate all of the results and models from this study are available at: https://github.com/AstraZeneca/Multimodal_NSCLC.
