# Supplementary material for: Autoencoder-based multimodal prediction of non-small cell lung cancer survival
Source: Sci Rep. 2023 Sep 22;13:15761. doi: 10.1038/s41598-023-42365-x (PMC10517020; doi:10.1038/s41598-023-42365-x)

Autoencoder-Based Multimodal Prediction of Non-Small Cell Lung Cancer Survival
Authors: Jacob Gould Ellen, Etai Jacob, Nikos Nikolaou, Natasha Markuzon

Supplementary Figures and Tables

Supplementary Table 1. Survival Performance for All Modality Combinations Stratified by Early and Late Integration of Biological Modalities

Data Combinations	LUAD			LUSC		
	C-Index (Early)	C-Index (Late)	Early-Late	C-Index (Early)	C-Index (Late)	Early-Late
lncRNA + Clinical	0.69 (± .03)	-	-	0.62 (± .03)	-	-
Gene + miRNA + Clinical	0.69 (± .02)	0.65 (± .04)	.04	0.56 (± 0.02)	0.56 (± .03)	0
Gene + lncRNA	0.68 (± .03)	0.65 (± .03)	.03	0.60 (± .03)	0.56 (± .03)	.04
lncRNA + miRNA	0.68 (± .02)	0.66 (± .03)	.02	0.55 (± .05)	0.58 (± .02)	-.03
Methylation + miRNA + Clinical	0.68 (± .03)	0.65 (± .03)	.03	0.60 (± .03)	0.60 (± .03)	0
Gene + Clinical	0.67 (± .03)	-	-	0.58 (± .03)	-	-
Methylation + Gene + Clinical	0.67 (± .03)	0.66 (± .03)	.01	0.61 (± .02)	0.59 (± .02)	.02
Methylation + Gene + lncRNA	0.67 (± .04)	0.64 (± .02)	.03	0.58 (± .03)	0.58 (± .02)	0
Methylation + lncRNA	0.67 (± .03)	0.63 (± .03)	.04	0.60 (± .03)	0.59 (± .02)	.01
Gene + miRNA + lncRNA + Clinical	0.66 (± .03)	0.67 (± .03)	-.01	0.61 (± .02)	0.57 (± .03)	.04
Methylation + Gene + lncRNA + Clinical	0.66 (± .04)	0.67 (± .03)	-.01	0.58 (± .02)	0.59 (± .03)	-.01
Methylation + Gene + lncRNA + miRNA	0.66 (± .03)	0.65 (± .02)	.01	0.61 (± .03)	0.57 (± .03)	.04
Methylation + Gene + miRNA	0.66 (± .04)	0.62 (± .03)	.04	0.58 (± .02)	0.56 (± .03)	.02
Methylation + Gene + miRNA + Clinical	0.66 (± .03)	0.66 (± .03)	0	0.59 (± .02)	0.60 (± .03)	-.01
Methylation + lncRNA + Clinical	0.66 (± .03)	0.66 (± .02)	0	0.57 (± .02)	0.62 (± .03)	-.05
Methylation + lncRNA + miRNA	0.66 (± .03)	0.63 (± .02)	.03	0.61 (± .03)	0.58 (± .03)	.03
Methylation + miRNA	0.66 (± .03)	0.57 (± .02)	.09	0.57 (± .03)	0.57 (± .03)	0
miRNA + lncRNA + Clinical	0.66 (± .03)	0.68 (± .03)	-.02	0.58 (± .03)	0.60 (± .02)	-.02
Gene + lncRNA + Clinical	0.65 (± .03)	0.67 (± .03)	-.02	0.57 (± .03)	0.57 (± .03)	0
Gene + miRNA	0.65 (± .03)	0.62 (± .02)	.03	0.57 (± .02)	0.55 (± .03)	.02
Gene + miRNA + lncRNA	0.65 (± .04)	0.65 (± .03)	0	0.59 (± .04)	0.56 (± .03)	.03
Methylation + lncRNA + Clinical + miRNA	0.65 (± .04)	0.66 (± .03)	-.01	0.59 (± .03)	0.61 (± .03)	-.02
Methylation + Clinical	0.64 (± .04)	-	-	0.58 (± .03)	-	-
Methylation + Gene	0.64 (± .03)	0.63 (± .02)	.01	0.56 (± .02)	0.58 (± .03)	-.02
miRNA + Clinical	0.63 (± .03)	-	-	0.56 (± .02)	-	-
**Average**	0.662	0.646	.016	0.585	0.580	.005
**Average (Modalities with Only Biological Data)**	0.662	0.632	.03	0.584	0.571	.013
**Average (Modalities with Clinical Data)**	0.662	0.659	.003	0.586	0.587	-.001

Supplementary Figure 1. Differential Expression Analysis Volcano Plots for lncRNA, miRNA and mRNA data showing significantly higher expressed genes (red) and lower expressed genes (blue) in survival subgroup G1 using a 0.05 p-value cutoff and a 0.58 Log2FoldChange cutoff

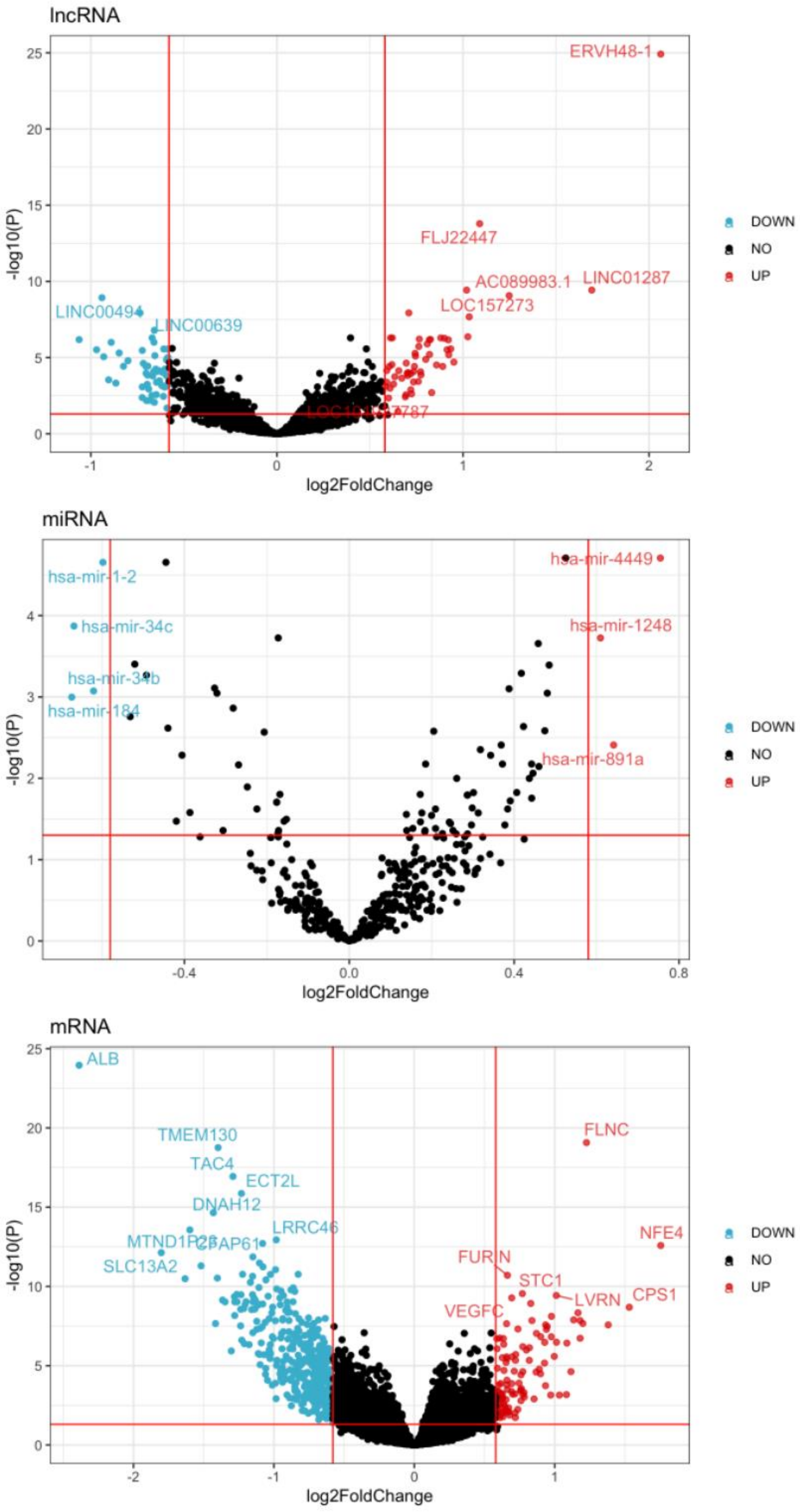

Supplementary Table 2. Comparing basic and denoising autoencoders (Gaussian and Zeros) by C-index and RMSE of input reconstruction for training/testing data

Autoencoder Type	Activation Function	Parameter[a]	Mean C-Index	Mean Train RMSE	Mean Test RMSE
**Denoising (Zeros)**	ReLU	0.2	0.587	0.560	0.639
	Sigmoid	0.2	0.584	0.507	0.600
	Tanh	0.2	0.582	0.515	0.610
	ReLU	0.3	0.583	0.686	0.682
	**Sigmoid**	**0.3**	**0.592**	0.518	0.611
	Tanh	0.3	0.583	0.535	0.621
	ReLU	0.4	0.589	0.894	0.775
	Sigmoid	0.4	0.588	0.530	0.624
	Tanh	0.4	0.576	0.559	0.637
Mean (± SE)	-	-	0.585 (± .005)	0.589 (± .126)	0.644 (± .055)
Denoising (Gaussian)	ReLU	0.1	0.590	0.574	0.746
	Sigmoid	0.1	0.584	0.813	0.827
	Tanh	0.1	0.575	0.566	0.632
	ReLU	0.5	0.588	0.570	0.729
	Sigmoid	0.5	0.581	0.816	0.828
	Tanh	0.5	0.586	0.567	0.615
	ReLU	1	0.579	0.611	0.746
	Sigmoid	1	0.578	0.823	0.833
	Tanh	1	0.582	0.591	0.632
Mean (± SE)	-	-	0.582 (± .005)	0.659 (± .120)	0.732 (± .088)
Basic	ReLU	-	0.581	0.536	0.661
	Sigmoid	-	0.587	0.699	0.713
	Tanh	-	0.579	0.587	0.702
Mean (± SE)	-	-	0.582 (± .003)	0.607 (± .068)	0.692 (± .022)

[a]The tuning parameters are the percentage of zero values for the denoising zeros autoencoder, the standard deviation of the distribution of noise for the denoising gaussian autoencoder, and there are no tuning parameters for the basic autoencoder.

Supplementary Table 3. Survival performance, as measured by mean C-index ± standard error, of the pipeline on LUSC and LUAD patients with different combinations of training data for each cancer type

**Training data**	**Testing data**	**miRNA**	**Methylation**	**lncRNA**	**mRNA**	**Clinical**	**Multimodal**	**Mean C-index**
LUSC	LUAD	0.59 ± 0.02	0.55 ± 0.02	0.61 ± 0.03	0.57 ± 0.03	0.60 ± 0.02	0.57 ± 0.03	0.58 ± 0.01
	LUSC	0.51 ± 0.05	0.50 ± 0.04	0.54 ± 0.03	0.57 ± 0.03	0.54 ± 0.04	0.55 ± 0.03	0.54 ± 0.01
LUAD	LUAD	0.62 ± 0.03	0.55 ± 0.05	0.65 ± 0.04	0.62 ± 0.03	0.64 ± 0.03	0.65 ± 0.04	0.62 ± 0.02
	LUSC	0.50 ± 0.03	0.55 ± 0.04	0.59 ± 0.04	0.49 ± 0.03	0.59 ± 0.03	0.54 ± 0.04	0.54 ± 0.02
LUSC + LUAD	LUAD	0.60 ± 0.02	0.57 ± 0.05	0.64 ± 0.03	0.63 ± 0.03	0.64 ± 0.03	0.67 ± 0.04	**0.63 ± 0.01**
	LUSC	0.54 ± 0.04	0.55 ± 0.03	0.56 ± 0.02	0.55 ± 0.03	0.59 ± 0.03	0.59 ± 0.03	**0.56 ± 0.01**

Supplementary Table 4. Comparison of models' performance using early versus late integration of modalities

NSCLC type	C-index (mean ± SE)	
	**Late integration**	**Early integration**
LUAD	0.67 ± 0.04	0.67 ± 0.04
LUSC	0.59 ± 0.03	0.63 ± 0.02

Supplementary Figure 2. Feature selection and autoencoder-based feature reduction methods applied for early and late multimodal data integration

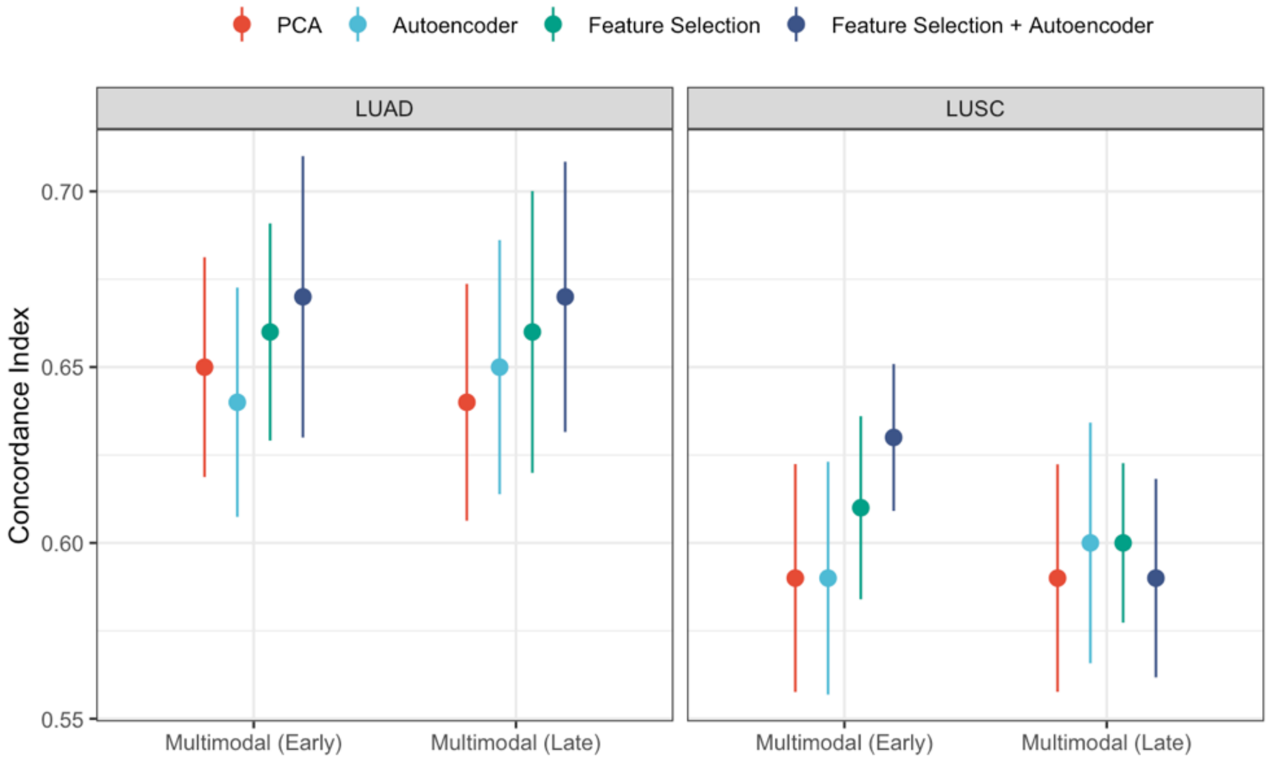

Supplementary Table 5. Number of total data points and patients for each data modality

Data Type	Number of Initial Datapoints	LUAD Cases	LUSC Cases	Total NSCLC Cases
miRNA	1,881	519	478	997
mRNA	44,162	533	502	1035
DNA Methylation	485,512	479	370	849
lncRNA	12,440	533	502	1035
Clinical Data	11	533	502	1035
Total	544,006	408	324	732[a]

[a]Final Number of Patients after taking only overlapping cases and data preprocessing

Supplementary Figure 3. General structure of the denoising autoencoder in this study for all biological modalities combined (early integration). The 11 clinical features were added later to form a 171-dimensional vector used for survival predictions.

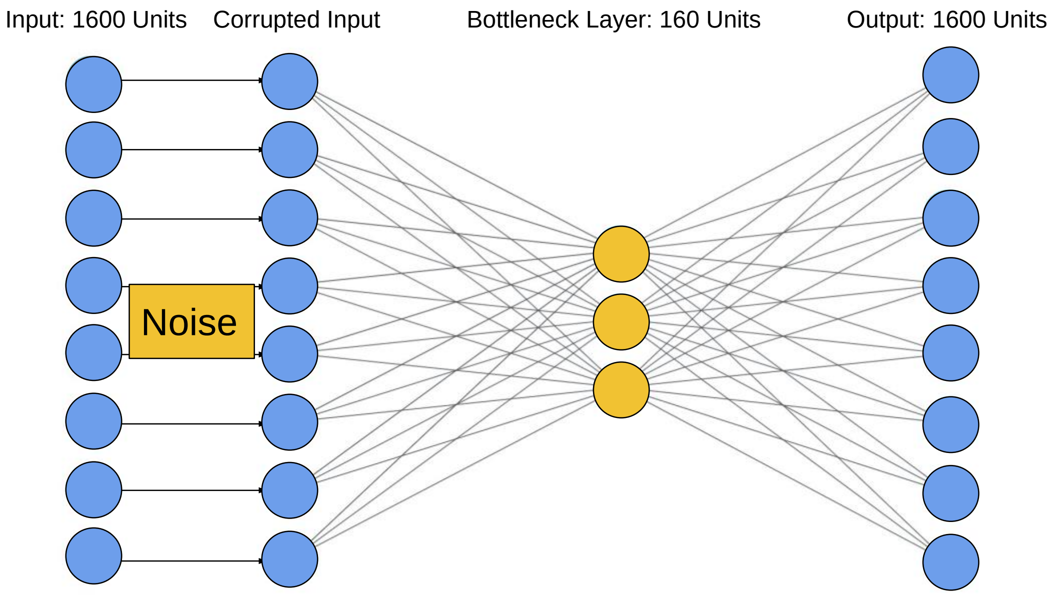

Supplement: Supplementary file 1 — Supplementary Information. [file 41598_2023_42365_MOESM1_ESM.pdf]
